# A higher response of plasma neuropeptide Y, growth hormone, leptin levels and extracellular glycerol levels in subcutaneous abdominal adipose tissue to Acipimox during exercise in patients with bulimia nervosa: single-blind, randomized, microdialysis study

**DOI:** 10.1186/1743-7075-8-81

**Published:** 2011-11-17

**Authors:** Kvido Smitka, Hana Papezova, Karel Vondra, Martin Hill, Vojtech Hainer, Jara Nedvidkova

**Affiliations:** 1 Institute of Endocrinology, Laboratory of Clinical and Experimental Neuroendocrinology, Narodni 8, 116 94 Prague 1, Czech Republic; 2 Psychiatric Clinic, First Faculty of Medicine, Charles University, Ke Karlovu 11, 121 08 Prague 2, Czech Republic

**Keywords:** Neuropeptide Y, Growth hormone, Leptin, Bulimia nervosa, Acipimox, Exercise, Adipose tissue, Free fatty acids, Glycerol, Microdialysis

## Abstract

**Background:**

Neuropeptide Y (NPY) is an important central orexigenic hormone predominantly produced by the hypothalamus, and recently found to be secreted in adipose tissue (AT). Acipimox (Aci) inhibits lipolysis in AT and reduces plasma glycerol and free fatty acid (FFA) levels. Exercise and Aci are enhancers of growth hormone (GH) and NPY secretion and exercise may alter leptin levels. We expect to find abnormal neuropeptidergic response in plasma and AT in patients with bulimia nervosa (BN). We hypothesize that Aci influences these peptides via a FFA-independent mechanism and that Aci inhibits lipolysis through a cyclic adenosine monophosphate (cAMP)-dependent pathway. Dysregulations of the AT-brain axis peptides might be involved in binge eating as is the case in BN.

**Methods:**

The objective of this study was to determine the responses of plasma NPY, GH, leptin, FFA and glycerol levels to exercise in BN patients and healthy women (C) given the anti-lipolytic drug Aci or placebo. The secondary objective of this study was to compare the responses of extracellular glycerol levels and plasma glycerol levels to exercise alone or together with Aci administration in BN patients and C women. Extracellular glycerol was measured *in vivo *in subcutaneous (sc) abdominal AT using microdialysis. Eight BN and eight C women were recruited for this single-blind, randomized study. Aci or placebo was given 1 hour before the exercise (45 min, 2 W/kg of lean body mass [LBM]). NPY, GH, leptin, FFA, glycerol plasma and AT glycerol levels were measured using commercial kits.

**Results:**

The primary outcome of this study was that the exercise with Aci administration resulted in plasma NPY and GH increase (after a 45-minute exercise) and leptin (after a 90-minute post-exercise recovering phase) increased more in BN patients. The secondary outcomes of this study were that the exercise with Aci administration induced a higher decrease of extracellular glycerol in BN patients compared to the C group, while the exercise induced a higher increase of glycerol concentrations in sc abdominal AT of BN patients. Plasma glycerol levels decreased more in BN patients and plasma FFA levels were depressed in both groups after the exercise with Aci administration. The exercise induced similar increases in plasma NPY, GH, FFA and glycerol levels, and a similar decrease in the plasma leptin level in both groups.

**Conclusions:**

We confirm the results of a single-blind, randomized, microdialysis study, i.e. that the Aci-induced elevation in plasma NPY and GH levels during the exercise is higher in BN patients and that Aci increased plasma leptin levels in the post-exercise recovering phase (90-minute) more in BN patients. The post-exercise rise (45-minute) in AT glycerol is much more attenuated by acute Aci treatment in BN patients. Simultaneously, we found facilitated turnover of plasma glycerol after the exercise together with Aci administration in BN. Our results support the hypotheses that Aci exerts an effect on the FFA-independent and cAMP-dependent mechanism.

**Trial Registration:**

Australia and New Zealand Clinical Trials Register (ANZCTR): ACTRN12611000955910

## Background

Bulimia nervosa (BN) is an eating disorder characterized by repeated episodes of binge eating followed by inappropriate compensatory behavior with no pathological change in body weight, leading to so far poorly understood neuroendocrine dysfunction of the hypothalamic-pituitary-adrenal axis [1]. Neuropeptide Y (NPY) is expressed predominantly in the neurons of the hypothalamic arcuate nucleus and co-exists with catecholamines in the central and the sympathetic nervous system (SNS), which play an important role in the up- or down-regulation of adipose tissue (AT) lipolysis [2]. Moreover, NPY receptors are highly expressed on human adipocytes where they inhibit lipolysis [3]. Recently, it was found that NPY is synthesized in human AT and stimulates proliferation and differentiation of new adipocytes [4], and that adipose-derived NPY may have implications for central feedback of adiposity signals.

Both fasting and exercise are catabolic stress states during which secretion of growth hormone (GH) increases and activation of the GH secretagogue receptors (GHSR), abundantly expressed in the arcuate nucleus, up-regulates expression of NPY [5,6]. It has been reported that NPY can attenuate specific behavior when the organism is stressed and anti-stress effects of NPY are relevant to psychiatric conditions such as anorexia nervosa (AN) and BN [7,8].

Physical exercise and anti-lipolytic drugs are well-recognized stimulators of several hormones, such as GH and NPY [5,9,10], however, molecular mechanisms and receptor array underlying these regulations are different and remain unidentified [10-14]. Even though plasma NPY levels do not reflect NPY secretion in the brain, there is not a clear evidence that plasma NPY levels originate from peripheral sympathetic nerve secretion or the adrenal gland and/or AT during exercise in humans [15].

In our recent study, we reported increased response of GH and ghrelin to exercise and anti-lipolytic drug administration in BN patients, and we confirmed that GH exerted an inhibitory feedback effect on plasma ghrelin during exercise only in BN patients; however, this effect was exerted in both BN patients and healthy women during exercise with Acipimox (Aci) administration [16].

Recent experimental evidence suggests that plasma NPY is increased in low plasma leptin states, and that plasma leptin levels are negatively correlated with NPY in patients with AN [17]. Furthermore, leptin is involved in the control of glucose-lipid homeostasis, energy storage and multiple neuroendocrine functions, which are impaired in BN and AN patients, including the pathological adaptation of the organism to starvation [18,19]. It is possible that the malnutrition-dependent reduction of leptin levels may play a role in the hypersomatotropism of AN [20,21]. This GH secretory pattern, which may reflect an adaptive phenomenon, is favoring the preferential utilization of free fatty acids (FFA). Indeed, we have previously shown that anorectic patients have a higher rate of lipolysis relative to healthy women [22]. It has been suggested that fasting and exercise-enhanced appetite and food intake are due to an increased negative energy balance, causing a change of the levels of some hypothalamic and extra-hypothalamic peptides such as NPY. Karamouzis et al [9] examined the effect of intense prolonged exercise on plasma leptin and NPY levels in man. This indicates that low level of leptin facilitates expression of NPY synthesis. The effect of exercise on decreased plasma leptin is suggested via increased plasma FFA levels [23], although direct action of FFA on plasma leptin levels cannot be concluded [24]. However, leptin production was stimulated by the anti-lipolytic agent Aci from isolated rat adipocytes [25]. On the basis of our previous study [16] we expect to find abnormal neuropeptidergic response in plasma and AT in BN patients. We hypothesize that plasma NPY, GH and leptin levels induced by the exercise together with Aci administration are not mediated by FFA.

Leptin-NPY-GH interactions and the role of appetite-regulating neuropeptides in mediating lipolysis in humans are not well understood. Dysregulations of the AT-gut-brain axis peptides might be involved in the pathogenesis of BN patients [16,26,27]. In this eating disorder characterized by repeated episodes of binge eating followed by inappropriate compensatory behavior, increased baseline plasma GH and NPY levels were documented, similarly as in restrictive-type AN patients with low body mass index (BMI) [20,28,29].

Aci is a potent and long-acting anti-lipolytic drug, derived from niacin. The inhibition of adipocyte lipolysis by Aci is mediated through suppression of intracellular cyclic adenosine monophosphate (cAMP) levels, which inhibit adipocyte lipases such as hormone-sensitive lipase (HSL) and the adipose triglyceride lipase (ATGL, i.e. desnutrin) via alternative cAMP-independent pathways [25,30-32], thereby lowering circulating plasma FFA and glycerol levels. We hypothesize that Aci influences inhibition of adipocyte lipolysis via the cAMP-dependent mechanism.

The primary aim of this study was to examine the changes in plasma NPY, GH, leptin, insulin, blood glucose, FFA and glycerol levels induced by physical exercise alone or in association with anti-lipolytic drug Aci administration in BN patients and healthy women. The secondary aim of this study was to compare the responses of AT glycerol and plasma glycerol to exercise alone or together with Aci administration in BN patients. Healthy women were used as the control group.

## Subjects and Methods

The study was performed in accordance with the Declaration of Helsinki and was approved by the Ethics Committee of the Institute of Endocrinology in Prague. Each participant signed an informed consent form before entering the study. The study took place in the Laboratory of Clinical nd Experimental Neuroendocrionology of the Institute of Endocrinology in Prague from January 2008 to June 2009. The study is registered at Anzctr.org.au, ACTR Number: ACTRN12611000955910.

### Patients and healthy women

Eight women with BN (means ± S.E.M., age: 24.33 ± 1.38 years; BMI: 20.63 ± 0.80 kg/m^2^; percentage of body fat [% BF]: 24.83 ± 1.92) and eight healthy women (age: 25.83 ± 1.69 years; BMI: 19.98 ± 0.44 kg/m^2^; % BF: 24.5 ± 0.47) were recruited for this study. All subjects included in the study were nonsmokers, had no allergies and had been free of medications for at least two weeks prior to the study. Healthy volunteers had no history of cardiovascular disease, eating disorders or other psychiatric diseases. All healthy women were in the first two weeks of the follicular phase of their menstrual cycle. Patients with BN were diagnosed according to the 4^th ^edition of the Diagnostic and Statistical Manual of Mental Disorders, American Psychiatric Association, 1994 [33]. All BN patients were clinically stable and in relatively good health, except for their eating disorder. In BN patients, the average frequency of binge-purging episodes was 2.5 times per day over the last three-month period and the average duration of their eating disorder was 6 years and 8 months. They were investigated after 1 week of hospitalization at the Department of Psychiatry of the Charles University, Prague. Main inclusion criteria were: age between 18 and 30 years, BMI between 18 and 23 kg/m^2^, patients with a diagnosis of BN [33]. Women with diabetes type 1 or 2, hypo- or hyperthyreoidism, cardiovascular disease, pregnant or lactating women, patients with any severe active infection or cancer, patients with impaired mental capacity or other psychiatric diseases were not eligible for participation in the study. Other exclusion criteria were: hypertension, abnormal blood tests with significant hyperlipidaemia, history or presence of hepatic or renal disorders. For two weeks before the study they had to refrain from taking anti-depressant and contraceptive drugs. For five days before study they had to refrain from taking aspirin, anti-histamine drugs and Tylenol (acetaminophen). Two days before study they had to avoid non herb tea, coffee (even decaffeinated), alcohol, chocolate, cocoa, nuts, and bananas, and avoid smoking. All subjects starved overnight with the exception of drinking water. From all bulimic patients (14) and healthy women (10), 8 BN patients and 8 healthy women were acceptable during the inclusion procedure (the recruitment phase in the Institute of Endocrinology) for being randomized, i.e. 3 BN patients were excluded, 1 BN patient did not meet inclusion criteria, 2 BN patients declined to participate in the study, 1 healthy woman did not meet inclusion criteria and 1 healthy woman declined to participate in this study. For allocation of all participants (8 BN patients and 8 healthy women) in the Institute of Endocrinology, a computer-generated list of random numbers was used. Our study used the allocation ratio 1:1 for two groups.

### Experimental protocol and blood sampling

Blood tests conducted before initiation of the study confirmed normal values for blood count, fasting blood glucose, and liver and renal function. All subjects consumed a standardized low-fat dinner (468 kcal [1955 kJ], 5-g fat, 70-g carbohydrate and 25-g protein) at 6:00 PM and were then refrained from eating overnight. Reported duration of sleep in the night preceding blood sampling was comparable in all studied subjects. Subjects were admitted to the Institute of Endocrinology at 7:00 AM. After a short medical examination (blood pressure, heart, and respiratory rate measurement, electrocardiogram [ECG]), % BF was estimated by anthropometric measurement and bioimpedancy (TANITA, Tokyo, Japan). Before starting the test, all individuals rested on the bed for 45 minutes. The placebo was matched to the study drug Aci for taste, color, and size, and contained microcrystalline cellulose, identical in appearance but without the active ingredient. Each woman was assigned a serial number and received four capsules in two bottles. All subjects were randomized according to a computer generated randomisation list to receive either placebo or Aci capsules each week (two 250 mg capsules of the acute-bolus Aci therapy or placebo; 500 mg total - 5-Methylpyrazine-2-carboxylic acid 4-oxide, molecular weight: 154.1, Olbetam capsules, Farmitalia Carlo Erba, Milan, Italy) 1 hour before a single exercise bout, once a week over a total of 2 weeks. A low- to moderate-intensity exercise bout on an electromagnetically braked bicycle ergometer (Cateye EC 1600, Japan) was performed for 45 min at power output 2 W/kg of lean body mass (LBM) and the average pedal speed was 60 revolutions per minute, intended to be below the aerobic-anaerobic threshold. ECG, heart rate and blood pressure were monitored using an Eagle 3000 cardiomonitor (Marquette, Milwaukee, WI, USA) and haemodynamic parametres were measured every 5 minutes during the 45-minute exercise bout. At 8:00 AM, after overnight fasting, a venous catheter was inserted into the antecubital vein. A blood sample was collected at the beginning and in the course (after 45-minute exercise, after a 90-minute post-exercise recovering phase) of the experiment to estimate plasma NPY, GH, leptin, FFA, glycerol, insulin and blood glucose concentrations. Blood samples were collected into chilled tubes containing Na_2_EDTA and antilysin. Plasma was separated immediately by centrifugation at 4°C and stored at -80°C until being assayed. Subjects started their 45-min exercise alone or exercise after Aci administration, assigned randomly 60 minutes before exercise once a week over a total of 2 weeks. After the 45-min exercise, all subjects assumed a resting supine position on a comfortable bed for additional 90 minutes.

### Microdialysate sampling

The *in situ *and *in vivo *microdialysis technique was used to examine the exercise-stimulated lipolysis (by measurement of dialysate glycerol) alone or with the acute-bolus Aci therapy randomly received (500 mg per os 1 hour before a single exercise bout, Olbetam capsules, Farmitalia Carlo Erba, Milan, Italy) once a week over a total of 2 weeks. A CMA-60 microdialysis probe (CMA Microdialysis, Stockholm, Sweden) with membrane length 3 cm and molecular weight cut-off 20 kDa was inserted sc under sterile conditions (8-10 cm left of the umbilicus at least 60 min before microdialysate sampling). After insertion of the CMA-60 catheter, perfusion with sterile Ringer solution was started at a flow rate of 2 μl/min using a CMA 107 microdialysis pump (CMA Microdialysis, Stockholm, Sweden). Microdialysate samples were collected every 30-45 min over a 6-hour period, 120 min before the exercise (at 45 min interval-baseline values), 45 min during the exercise and at the last 45-min interval before ending the 90-min post-exercise recovering phase. The microdialysate volumes of samples measured at 45-min intervals were 88 ± 3 μl. Microvials were placed on ice immediatelly after the collection, and stored at -80°C until analysis. Before starting microdialysis perfusion, the relative glycerol recovery (RGR) was calculated *in vitro *at a temperature of 37°C maintained by a digital block heater to simulate body temperature. Different perfusion rates (0.1, 0.3, 0.5, 1.0, 2.0, 5.0 μl/min) were tested to investigate possible relative recovery vs. perfusion rate dependency. At each rate, RGR was calculated from 15 samples collected at perfusion rate-dependent intervals according to the formula: RGR (%) = (glycerol concentration in dialysate/glycerol concentration in standard solution) × 100. A perfusion rate of 2 μl/min was selected for *in vivo *microdialysis, based on the calculated *in vitro *RGR corrected for experiment duration. The procedure is described in detail in our previous report [34].

### Hormonal and biochemical assays

NPY in plasma (200 μl) was determined by a commercial RIA kit (Linco Research, Inc., St. Charles, Missouri, USA). The intra- and interassay variability for plasma NPY was 5.0% and 8.4%, respectively, sensitivity was 3 pmol/l. GH in plasma (50 μl) [as a 22 kDa monomeric GH form and non-22 kDa isoforms (dimers and GH bound to plasma proteins)] was measured by a commercial RIA kit (Immunotech, Prague, Czech Republic). Intra- and inter-assay variability was 1.5% and 14%, respectively, sensitivity was 0.1 μIU/ml. Leptin in plasma (200 μl) was measured by a commercial RIA kit (Linco Research, St. Charles, Missouri, USA). Sensitivity, inter-assay and intra-assay variability were 0.05 ng/ml, 8.6% and 5.9%, respectively. Insulin in plasma (10 μl) and blood glucose in plasma (10 μl) were measured in a Cobas Integra 400 plus system (Roche Diagnostics, GmbH, Mannheim, Germany). Glycerol in plasma (10 μl) and in the dialysate (10 μl) was analyzed with a radiometric kit (Randox Laboratories, GY 105, Montpellier, France). FFA in plasma (10 μl) were estimated colorimetrically with a commercial kit (Randox Laboratories, FA 115, Montpellier, France). All assays were run twice in duplicate.

### Statistical analysis

All values are presented as the means ± S.E.M. All statistical comparisons were performed using General Linear Model consisting of subject factor, between-subject factor Status, within-subject factors Aci and Time and interactions Status × Aci and Status × Time. Correlations between parameters were examined using Spearman's rank correlation coefficient. The original data was transformed by power transformations (individually for each dependent variable) to attain Gaussian's data distribution and constant variance in the data and residuals. The homogeneity of the data after power transformations was tested by residual analysis. Absolute values of Studentized residuals in transformed data were less than 3 for all variables and all experimental points. The difference between medians (Mann-Whitney and Wilcoxon Rank-Sum tests) was applied to compare baseline values with those during exercise. A *P *value < 0.05 denoted statistical significance.

## Results

### Tables and figures

**Figure 1:**
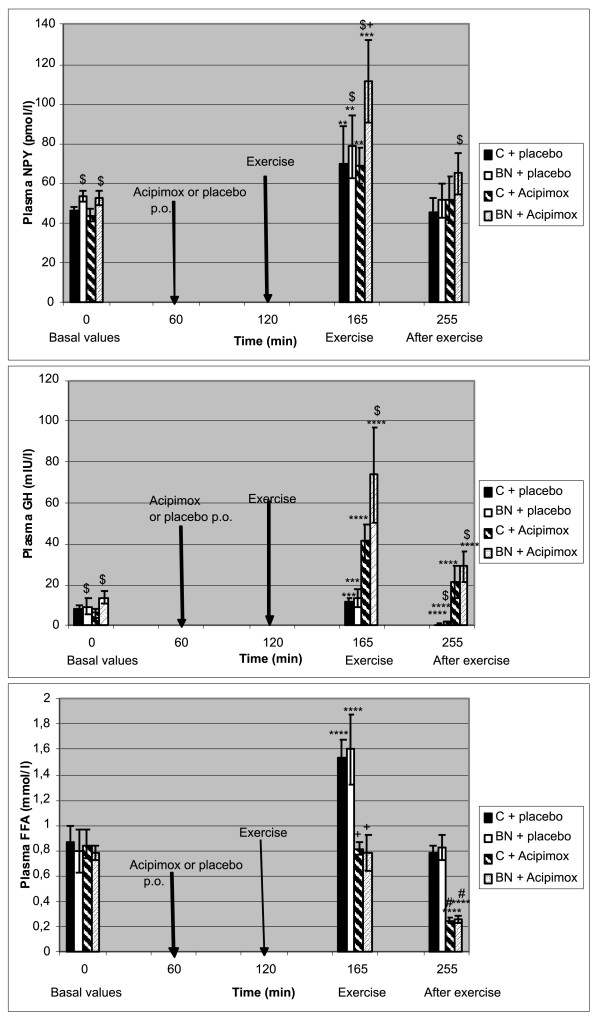
**Effect of exercise alone or together with Acipimox administration on plasma NPY, GH and FFA levels**. ^$^*P *< 0.05 vs. control subjects (C), p.o., per os. **P < 0.01, ***P < 0.001, ****P < 0.0001 vs. resting (baseline) values. ^+^*P *< 0.05 exercise together with Aci administration vs. exercise alone, 45 minutes. ^#^*P *< 0.05 post-exercise recovering phase together with Aci administration vs. post-exercise recovering phase alone, 90 minutes. Effect of 45-min exercise (2 W/kg of lean body mass [LBM]) alone or together with Acipimox (Aci) administration on plasma neuropeptide Y (NPY), growth hormone (GH) and free fatty acids (FFA) levels (means ± S.E.M.) in the controls (C) (*n *= 8) and bulimia nervosa (BN) patients (*n *= 8).

**Figure 2:**
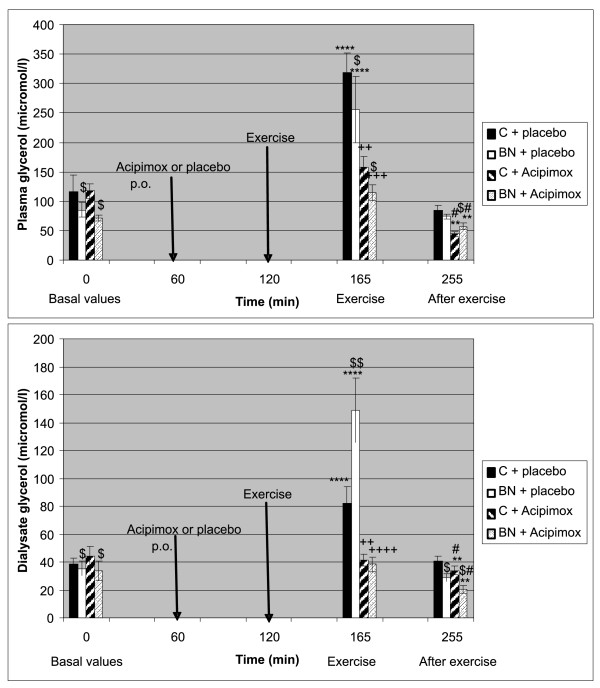
**Effect of exercise alone or together with Acipimox administration on plasma and dialysate glycerol levels**. **^$^***P ***<**0.05 BN vs. control subjects (C), p.o., per os. ^$$^*P *< 0.01 BN vs. control subjects (C). **P < 0.01, ****P < 0.0001 vs. resting (baseline) values. ^+ +^*P *< 0.01 exercise together with Aci administration vs. exercise alone, 45 minutes. ^+++^*P *< 0.001 exercise together with Aci administration vs. exercise alone, 45 minutes. ^++++^*P *< 0.0001 exercise together with Aci administration vs. exercise alone, 45 minutes. ^#^*P *< 0.05 post-exercise recovering phase together with Aci administration vs. post-exercise recovering phase alone, 90 minutes. Effect of 45-min exercise (2 W/kg of lean body mass [LBM]) alone or together with Acipimox (Aci) administration on plasma glycerol and dialysate glycerol levels (means ± S.E.M.) in the controls (C) (*n *= 8) and bulimia nervosa (BN) patients (*n *= 8).

**Figure 3:**
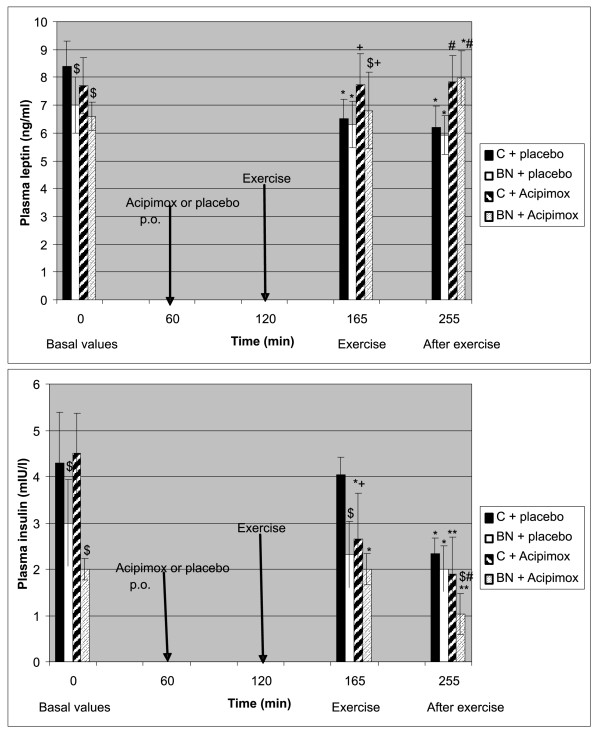
**Effect of exercise alone or together with Acipimox administration on plasma leptin and insulin levels**. ^$^*P *< 0.05 vs. control subjects (C), p.o., per os. *P < 0.05, **P < 0.01 vs. resting (baseline) values. ^+^*P *< 0.05 exercise together with Aci administration vs. exercise alone, 45 minutes. ^#^*P *< 0.05 post-exercise recovering phase together with Aci administration vs. post-exercise recovering phase alone, 90 minutes. Effect of 45-min exercise (2 W/kg of lean body mass [LBM]) alone or together with Acipimox (Aci) administration on plasma leptin and insulin levels (means ± S.E.M.) in the controls (C) (*n *= 8) and bulimia nervosa (BN) patients (*n *= 8).

Baseline characteristics of the study subjects, including anthropometric measurements, are summarized in Table 1. The exercise-induced changes of the study subjects during Aci and placebo treatment in plasma and in AT are shown in Table 2 Table 3 Figure 1, Figure 2 and 3, respectively.

**Table 1:** Anthropometric characteristics of the study subjects (means ± S.E.M.).

	C (*n *= 8)	BN (*n *= 8)	*P *value
**Age (years)**	25.83 ± 1.69	24.33 ± 1.38	NS

**BMI (kg/m^2^)**	19.98 ± 0.44	20.63 ± 0.80	NS

**% BF**	24.50 ± 0.47	24.83 ± 1.92	NS

C = controls (n = 8); BN = bulimia nervosa (n = 8); BMI = body mass index; % BF = percentage of body fat; NS = not significant; *n *= the number of subjects.

**Table 2:** Effect of exercise alone or together with Acipimox administration on plasma hormones and biochemical parameters.

	0 min	45 min	45 min	90 min	90 min
	**Basal**	**Exercise**	**Exercise**	**Post-exercise**	**Post-exercise**

		**+ placebo**	**+ Aci**	**+ placebo**	**+ Aci**

**NPY **(pmol/l)					

C group (*n *= 8)	45 ± 2.3	69.7 ± 19.2**	69 ± 9.2**	45.6 ± 6.7	51.5 ± 12

BN group (*n *= 8)	53 ± 3.0^$^	78.3 ± 15.7**^$^	111.4 ± 20.6***^$^^+^	51.2 ± 8.6	65 ± 10.6^$^

**GH **(mIU/l)					

C group (*n *= 8)	7.1 ± 0,5	11.3 ± 2.2***	40.9 ± 8.7****	0.7 ± 0.2****	21.4 ± 8.1****

BN group (*n *= 8)	11.2 ± 0.9^$^	13.1 ± 4.3***	73.7 ± 23.1****^$^	2.01 ± 0.5****^$^	28.9 ± 7.5****^$^

**Leptin **(ng/ml)					

C group (*n *= 8)	7.83 ± 1.3	6.52 ± 0.68*	7.73 ± 1.13^+^	6.21 ± 0.75*	7.84 ± 0.94^#^

BN group (*n *= 8)	6.96 ± 1.1^$^	6.31 ± 0.83*	6.8 ± 1.38^$^^+^	5.93 ± 0.69*	7.98 ± 0.99*^#^

**Insulin **(mIU/l)					

C group (*n *= 8)	4.32 ± 0.98	4.05 ± 0.37	2.66 ± 0.98*^+^	2.33 ± 0.34*	1.9 ± 0.8**

BN group (*n *= 8)	2.5 ± 0.59^$^	2.32 ± 0.71^$^	2.0 ± 0.33*	2.01 ± 0.5*	1.03 ± 0.44**^$#^

**Glucose **(mmol/l)					

C group (*n *= 8)	4.72 ± 0.11	4.8 ± 0.26	4.63 ± 0.19	4.59 ± 0.15	4.33 ± 0.23

BN group (*n *= 8)	4.33 ± 0.13	4.46 ± 0.38	5.03 ± 0.34*^+^	4.07 ± 0.3	4.43 ± 0.26

**FFA **(mmol/l)					

C group (*n *= 8)	0.86 ± 0.3	1.54 ± 0.13****	0.82 ± 0.05^+^	0.79 ± 0.05	0.25 ± 0.02****^#^

BN group (*n *= 8)	0.79 ± 0.3	1.6 ± 0.28****	0.79 ± 0.14^+^	0.83 ± 0.1	0.26 ± 0.02****^#^

**Glycerol **(μmol/l)					

C group (*n *= 8)	117 ± 33	318 ± 34****	157.8 ± 18.4^++^	85 ± 7.3	44 ± 4.7**^#^

BN group (*n *= 8)	82.2 ± 26^$^	256 ± 56****^$^	114 ± 13^$+++^	74 ± 4.2	57 ± 6.2**^$#^

Values are means ± S.E.M. C = controls (*n *= 8), BN = bulimia nervosa (*n *= 8), *n *= the numbers of subjects are in brackets.

* = *P *< 0.05, ** = *P *< 0.01, *** = *P *< 0.001, **** = *P *< 0.0001 vs. resting (baseline) values

**^$ ^**= *P *< 0.05 BN vs. control subjects (C)

^+ ^= *P *< 0.05 exercise together with Aci administration vs. exercise alone, 45 minutes

^+ + ^= *P *< 0.01 exercise together with Aci administration vs. exercise alone, 45 minutes

^+++ ^= *P *< 0.001 exercise together with Aci administration vs. exercise alone, 45 minutes

^# ^= *P *< 0.05 post-exercise recovering phase together with Aci administration vs. post-exercise recovering phase alone, 90 minutes

Effect of 45-min exercise (2 W/kg of lean body mass [LBM]) alone or together with Acipimox (Aci) administration on plasma adipose tissue (AT)-gut-brain peptides, blood glucose, free fatty acids (FFA) and glycerol levels in the controls (C) (*n *= 8) and bulimia nervosa (BN) patients (*n *= 8).

**Table 3:** Effect of exercise alone or together with Acipimox administration on extracellular glycerol in adipose tissue.

	0 min	45 min	45 min	90 min	90 min
	**Basal**	**Exercise**	**Exercise**	**Post-exercise**	**Post-exercise**

		**+ placebo**	**+ Aci**	**+ placebo**	**+ Aci**

**Dialysate Glycerol **(μmol/l)					

C group (*n *= 8)	41.21 ± 4.43	82.2 ± 11.82****	41.6 ± 4.21^++^	40.5 ± 3.81	33.9 ± 3.16**^#^

BN group (*n *= 8)	36.39 ± 4.15^$^	148.6 ± 23.2****^$^^$^	38.3 ± 5.39^++++^	29.0 ± 2.9^$^	20.4 ± 3.01**^$#^

** = *P *< 0.01 vs. resting (baseline) values

**** = *P *< 0.0001 vs. resting (baseline) values

^$ ^= *P *< 0.05 BN vs. control subjects (C)

^$$ ^= *P *< 0.01 BN vs. control subjects (C)

^++ ^= *P *< 0.01 exercise together with Aci administration vs. exercise alone, 45 minutes

^++++ ^= *P *< 0.0001 exercise together with Aci administration vs. exercise alone, 45 minutes

^# ^= *P *< 0.05 post-exercise recovering phase together with Aci administration vs. post-exercise recovering phase alone, 90 minutes

Dialysate glycerol concentration in subcutaneous (sc) abdominal adipose tissue (AT) during baseline conditions (microvials with interstitial fluid samples were collected at 45-min intervals), during exercise (at 45-min interval, 2 W/kg of lean body mass [LBM]), and at the last 45-min interval before ending 90-min post-exercise recovering phase alone or together with Acipimox (Aci) administration in the controls (C) (*n *= 8) and bulimia nervosa patients (BN) (*n *= 8). Values are means ± S.E.M. *n *= the number of subjects.

### Correlations between parameters

#### The relationship of hormonal, biochemical and anthropometric parameters during basal conditions in patients with BN and healthy control women

Fasting plasma leptin levels correlated positively with BMI in the controls (*r *= 0.62, *P *= 0.03). Fasting plasma blood glucose concentrations correlated negatively with plasma FFA concentrations in BN (*r *= - 0.66, *P *= 0.02). Fasting plasma glycerol concentrations correlated positively with plasma FFA concentrations in BN patients (*r *= 0.69, *P *= 0.0001) and the controls (*r *= 0.87, *P *= 0.0001).

#### The relationship of hormonal and biochemical parameters after the exercise with Aci administration (45-minute) in patients with BN and in healthy control women

Plasma GH concentrations correlated positively with plasma NPY concentrations after 45-minute exercise with Aci administration only in BN patients (*r *= 0.56, *P *= 0.01). Plasma glycerol concentrations correlated positively with plasma FFA concentrations after 45-minute exercise with Aci administration in BN patients (r = 0.91, *P *= 0.004) and the controls (*r *= 0.93, *P *= 0.002). Plasma insulin levels correlated positively with plasma leptin levels after 45-minute exercise with Aci administration in the controls (*r *= 0.64, *P *= 0.04). Plasma leptin levels correlated positively with blood glucose concentrations after 45-minute exercise with Aci administration in the controls (*r *= 0.62, *P *= 0.03). Plasma FFA levels correlated positively with plasma insulin levels after 45-minute exercise with Aci administration in the controls (*r *= 0.68, *P *= 0.02).

#### Baseline and exercise-induced plasma NPY concentrations alone or together with Aci administration

Mean baseline fasting plasma NPY concentrations were significantly increased in BN patients compared to the controls Plasma NPY levels increased significantly after the 45-minute exercise with placebo in both groups, but the next week the administration of Aci 60 minutes before the 45-minute exercise led to a further increase of plasma NPY levels only in BN patients. Plasma NPY levels decreased to the baseline values after a 90-minute post-exercise recovering phase with placebo in both groups, but less in BN patients if associated with Aci administration compared to the controls (Table 2 Figure 1).

#### Baseline and exercise-induced plasma GH concentrations alone or together with Aci administration

Mean baseline fasting plasma GH concentrations were significantly increased in BN patients compared to the controls. Plasma GH concentrations increased significantly after the 45-minute exercise with placebo in both groups. The next week the administration of Aci 60 minutes before the 45-minute exercise increased plasma GH in both groups, more in BN patients. Plasma GH levels decreased significantly more in the controls after 90 minutes of post-exercise recovering phase with placebo than in BN patients. On the contrary, plasma GH levels were significantly elevated at 90 minutes after post-exercise recovering phase with Aci administration in both groups (Table 2 Figure 1).

#### Baseline and exercise-induced plasma FFA concentrations alone or together with Aci administration

Mean baseline fasting plasma FFA concentrations were similar in BN patients and the controls. Plasma FFA concentrations increased significantly after 45-minute exercise with placebo in both groups. Plasma FFA concentrations decreased significantly to the baseline levels after 45-minute exercise with Aci administration in both groups when compared to the exercise associated with placebo. Plasma FFA concentrations approached the baseline values after a 90-minute post-exercise recovering phase with placebo in both groups. Plasma FFA concentrations decreased significantly under the baseline values after 90 minutes of post-exercise recovering phase with Aci administration in both groups (Table 2 Figure 1).

#### Baseline and exercise-induced plasma glycerol concentrations alone or together with Aci administration

Mean baseline fasting plasma glycerol levels were significantly decreased in BN patients compared to the controls Plasma glycerol levels increased significantly after 45-minute exercise with placebo in both groups. Plasma glycerol concentrations approached the baseline values after 90 minutes of post-exercise recovering phase with placebo in both groups. Plasma glycerol levels decreased significantly after 45-minute exercise with Aci administration more in BN patients when compared to the exercise associated with placebo. Plasma glycerol concentrations significantly decreased under the baseline values after 90 minutes of post-exercise recovering phase with Aci administration in both groups (Table 2 Figure 2).

#### Baseline and exercise-induced AT glycerol concentrations alone or together with Aci administration

Mean baseline AT glycerol levels were significantly decreased in BN patients compared to the controls. AT glycerol concentrations increased significantly after 45-minute exercise with placebo more in BN patients. AT glycerol levels decreased significantly to the baseline values after 45-minute exercise with Aci administration in both groups when compared to the exercise associated with placebo. AT glycerol concentrations approached the baseline values after 90 minutes of post-exercise recovering phase with placebo in the controls, while AT glycerol concentrations decreased under the baseline values in BN patients. AT glycerol levels decreased significantly under the baseline levels after 90 minutes of post-exercise recovering phase with Aci administration in both groups (Table 3 Figure 2).

#### Baseline and exercise-induced plasma leptin concentrations alone or together with Aci administration

Mean baseline fasting plasma leptin levels were significantly decreased in BN patients compared to the controls. Plasma leptin levels decreased significantly after 45-minute exercise with placebo in both groups. Plasma leptin concentrations approached the baseline values after 45-minute exercise with Aci administration in both groups. Plasma leptin concentrations decreased significantly after 90 minutes of post-exercise recovering phase with placebo in both groups. Plasma leptin levels increased significantly after 90 minutes of post-exercise recovering phase with Aci administration in both groups when compared to the post-exercise recovering phase associated with placebo (Table 2 Figure 3).

#### Baseline and exercise-induced plasma insulin concentrations alone or together with Aci administration

Mean baseline fasting plasma insulin concentrations were significantly decreased in BN patients compared to the controls. Plasma insulin concentrations approached the baseline values after 45-minute exercise with placebo in both groups. The next week the administration of Aci 60 minutes before the 45-minute exercise decreased significantly plasma insulin in both groups. Plasma insulin levels decreased significantly after 90 minutes of post-exercise recovering phase with placebo in both groups. Plasma insulin levels decreased significantly after 90 minutes of post-exercise recovering phase with Aci administration more in BN patients (Table 2 Figure 3).

#### Baseline and exercise-induced plasma blood glucose concentrations alone or together with Aci administration

Mean baseline fasting plasma blood glucose concentrations were similar in BN patients and the controls. Plasma blood glucose levels were over the baseline values after 45-minute exercise with placebo in both groups. Plasma blood glucose levels increased significantly after 45-minute exercise with Aci administration in BN patients. Plasma blood glucose levels were under the baseline values after 90 minutes of post-exercise recovering phase with placebo in both groups. Plasma blood glucose levels approached the baseline values after 90 minutes of post-exercise recovering phase with Aci administration in both groups (Table 2).

## Discussion

In the present study, basal fasting NPY and GH plasma levels were increased and independent of BMI in BN patients, and were similarly increased after acute exercise (45-minute) with placebo in both groups. The important finding of the present study is that anti-lipolytic drug Aci administered 60 minutes before initiating short-term exercise induced further important increase of plasma NPY levels only in BN patients, and further increase of plasma GH levels in both groups, more in BN patients. Further, we found that basal fasting leptin plasma levels decreased in BN patients, and were similarly decreased after acute exercise (45-minute) with placebo in both groups. Importantly, anti-lipolytic drug Aci during short-term exercise not only prevented any falling of plasma leptin levels in both groups, but even increased plasma leptin in the post-exercise recovering phase (90 minutes) with Aci administration, more in BN patients.

Orexigenic hypothalamic peptide NPY participates in leptin, ghrelin and GH regulation pathways [35]. The molecular mechanisms by which acute exercise or anti-lipolytic drugs could cause an increase in plasma NPY and GH are not clear. It was demonstrated that ghrelin action can be modulated by NPY [36,37]. Until now, the neuroendocrine control of NPY secretion during exercise is still unknown. Thus, induced changes in NPY and GH may be working through divergent signal transduction pathways or receptor array [10,11,38-42]. Very recently, Coiro et al [43] reported that a somatostatinergic pathway is involved in the mechanism connecting physical exercise to NPY secretion.

In our present study, exercise induced an increase in plasma NPY levels in the controls and in BN women in whom the NPY increase was higher, without exhibiting any significant dependence on plasma leptin and ghrelin levels and on plasma glycerol and FFA concentrations [16]. These results may indicate a disorder of the gut-hypothalamic-AT pathway in these patients in order to prevent energy losses. Hence, post-exercise decrease of leptin and increase of NPY are probably part of adaptive mechanisms leading to conservation of energy storage in BN [16,44].

The exercise plus Aci-administration further increased plasma NPY and GH levels more in BN patients. Thus, elevated NPY and GH levels induced by exercise together with Aci do not appear to be directly mediated via FFA, and to influence ghrelin secretion in both groups, indicating that FFA probably are not ghrelin enhancers [16]. Interestingly, plasma GH levels after 45-minute exercise with Aci administration positively correlated with plasma NPY levels only in BN patients and these observations lead us to suggesting that GH can be responsible for the increase of NPY production [38].

Kos et al [4] demonstrated that NPY is expressed and secreted by human adipocytes. Moreover, NPY is co-localizated with norepinephrine in perivascular sympathetic nerves, and it can be assumed that the exercise-induced increase in plasma NPY can be associated with increased norepinephrine in AT [22]. These findings are in accordance with previous and recent reports suggesting higher activity of SNS in AT and disrupted adrenergic regulation of lipolysis observable in both receptor and postreceptor levels in sc abdominal AT in AN [45,46]. It has been shown by numerous studies that SNS can exert tonic inhibitory action on leptin secretion, and that adrenergic regulation may contribute to rapid decrease both of plasma insulin and leptin levels during exercise [44,47].

In the present study, we found significantly decreased both baseline plasma leptin and insulin levels in BN patients similarly as documented in AN patients [44,48]. Plasma leptin levels were significantly lower immediately after exercise (45-minute) and after the post-exercise recovering phase (90-minute) combined with placebo in both groups. On the other hand, plasma FFA levels were significantly increased immediately after exercise (45-minute), and after the post-exercise recovering phase (90-minute) associated with placebo the values approached the baseline ones in both groups. This may provide evidence that FFA are not involved in the exercise-induced leptin decrease. Furthermore, after the post-exercise recovering phase (90-minute) with placebo, a decrease in plasma insulin levels was observed in both groups.

The role of plasma leptin and FFA levels in exercise has not been defined. A negative correlation was found between plasma FFA and leptin levels [23] and it was suggested that lipolysis may explain the rapid leptin decrease after the exercise, although Gomez-Merino et al [47] failed to find any correlation between plasma leptin levels and plasma FFA levels after physical activity in humans. Moreover, treatment with Aci increased significantly plasma leptin levels in humans [49,50]. These observations led us to suggest that administration of Aci might prevent the drop of plasma leptin levels during short-term exercise in both groups; in addition, after the 90-minute post-exercise recovering phase with Aci administration plasma leptin levels increased significantly more in BN patients.

After the 45-minute exercise, GH and blood glucose concentrations were more expressed in the presence of Aci vs. placebo in BN patients, and plasma insulin levels were lower after the post-exercise recovering phase (90-minute) combined with Aci administration, more in BN patients. It has been shown that anti-lipolytic Aci may decrease insulin and increase blood glucose concentrations [51]. Thus, GH and Aci administration during exercise promote glucose production, and increased plasma blood glucose may also stimulate leptin secretion [50]. In our present study, plasma leptin levels correlated positively with blood glucose concentrations after 45-minute exercise with Aci administration in the controls. Likewise, Lissett et al [52] demonstrated that a single bolus of GH increases plasma leptin levels in humans.

Furthermore, we observed lower levels of both baseline AT and plasma glycerol in patients with BN when compared to age- and weight-matched healthy women. Some authors did not observe changed baseline plasma and AT glycerol concentrations in AN patients with low BMI compared to the controls [22,44] but others found higher local glycerol concentrations in AT of underweight patients with AN [46]. Likewise, under *in vivo *conditions, we found that the sensitivity of beta-adrenergic receptors to norepinephrine in sc abdominal AT is decreased in patients with AN. This may be due to changed SNS in sc abdominal AT that results in down-regulation of beta-receptors and therefore to decreased lipolysis to protect fat stores from further depletion by increased sympathetic nervous activity [22]. In agreement with this report, Dostalova et al [53] revealed normal dialysate leptin concentrations in sc abdominal AT in AN patients. One explanation of this may be a reduced efficiency of both the SNS and NPY inhibiting adipocyte leptin production in AN.

The lipolytic effect of GH is one of the most important actions of GH favoring the use of FFA as an energy source during exercise. GH increases lipolysis by both the HSL and indirectly, lowering the ability of adipocytes to respond to alpha_2_-adrenergic dependent inhibition of cAMP production [54]. Importantly, we found a discrepancy between overall (plasma) glycerol and local (dialysate) glycerol levels in BN patients, and we determined a significantly higher dialysate glycerol level during exercise in BN compared to the controls. Currently, it is well known that local (tissue) lipolysis does not reflect plasma glycerol levels during exercise in BN patients [22,45]. This discrepancy could be possibly explained by the fact that plasma glycerol concentration reflects the net amount of this parameter released from different sources, whereas dialysate glycerol concentration determines the quantity released in AT. Aci acutely received during the exercise led to much more abolished lipolysis in sc abdominal AT in BN than in the controls, which leads us to suggesting that altered lipolysis in BN may result from local modification of both adrenergic and NPY-ergic activities. Interestingly, the effect of Aci administration on higher epinephrine secretion was observed in obese and lean males [51]. Thus, Aci, catecholamines and NPY act via their inhibition of cAMP production in AT, rather than via alternative cAMP-independent pathways [25,30-32], and up-regulation of receptor subtypes and/or their sensitivity or affinity are much more effective in abolishing lipolysis in BN.

Furthermore, we found even higher plasma glycerol levels after the exercise combined with Aci administration in the controls. These observations led us to suggesting that glycerol is not easily remetabolized, hence stays behind and its level therefore increases, and that the decrease of plasma FFA levels to basal values is exerted by facilitated turnover of FFA in BN. Unexpectedly, we did not confirm that increased plasma FFA levels inhibit lipolysis via a feedback mechanism [55].

## Conclusions

Taken together, it can be concluded, based on this single-blind, placebo-controlled, randomized, microdialysis study that anti-lipolytic drug Aci during exercise (45-minute) further increases plasma NPY, GH and leptin levels (after 90 minutes of post-exercise recovering phase) in BN patients and leads to lipolysis abolished to a much higher extent in sc abdominal AT in BN. Thus, it appears that bulimic patients are very sensitive to negative caloric balance and acute administration of Aci, and show hyperreactive responses both in NPY, GH and leptin; these data establish NPY, GH and leptin as valid biomarkers of BN [16,28,29,44]. Our results support the hypothesis that elevated NPY and GH levels induced by the exercise together with Aci administration thus do not appear to be directly mediated via FFA and that exercise- and Aci-induced leptin releases are not mediated by FFA. Thus, these observations lead us to suggesting that Aci exerts an effect on a FFA-independent mechanism. Altogether, our results support the hypothesis that Aci acts via its inhibition of cAMP production in sc abdominal AT, rather than via alternative cAMP-independent pathways. In addition, the exercise induced similar increases in plasma glycerol levels in both groups while the exercise with Aci administration resulted in a stronger plasma glycerol decrease in BN patients. Thus, plasma glycerol is not easily remetabolized, and the decrease of plasma glycerol after the exercise associated with Aci administration could be exerted by facilitated turnover of plasma glycerol, which would reflect abnormal metabolic status in this eating disorder. Lower basal lipolysis in AT in BN patients may be due to a protective mechanism that prevents the exhaustion of energy reserves.

Endocrine perturbations and a dysfunction within the FFA-leptin-NPY-GH system may also take part in the etiopathogenesis of either bulimia or AN. Better understanding of the role of NPY and leptin agonists or inhibitors and their interactions with adipocyte lipolysis and the GH neuroendocrine axis may provide an entirely new therapeutic approach in treatment of BN and AN patients who poorly respond to various pharmacological therapies.

## List of abbreviations

FFA: free fatty acids; BN: bulimia nervosa; AN: anorexia nervosa; C: controls; Aci: Acipimox; BMI: body mass index; % BF: percentage of body fat; LBM: lean body mass; AT: adipose tissue; sc: subcutaneous; ECG: electrocardiogram; NPY: neuropeptide Y; GH: growth hormone; GHSR: GH secretagogue receptors; SNS: sympathetic nervous system; cAMP: cyclic adenosine monophosphate; HSL: hormone-sensitive lipase; ATGL: adipose triglyceride lipase (i.e. desnutrin); RGR: relative glycerol recovery; NS: not significant; *n*: number of subjects; p.o: per os.

## Competing interests

The authors declare that they have no competing interests.

## Authors' contributions

Authors' contribution to the work: KS and JN - conception, design, and conduct of the study, interpretation and evaluation of the data; HP - selection and clinical evaluation of the patients (psychiatrist); KV and VH - selection and clinical evaluation of the study subjects; MH - statistical evaluation of the data. All authors read and approved the final manuscript.

## Authors information

Kvido Smitka, M.D., Ph.D.

Laboratory of Clinical and Experimental Neuroendocrinology, Institute of Endocrinology, Narodni 8, Prague 1, 116 94, Czech Republic, Tel: +420224905266, E-mail: KS: ksmitka@endo.cz

Prof. Hana Papezova, M.D., Ph.D.

Psychiatric Clinic, First Faculty of Medicine, Charles University, Ke Karlovu 11, Prague 2, 121 08, Czech Republic, E-mail: HP: hpap@seznam.cz

Prof. Karel Vondra, M.D., Ph.D.

Institute of Endocrinology, Narodni 8, Prague 1, 116 94, Czech Republic, E-mail: KV: kvondra@endo.cz

Martin Hill, Ph.D.

Institute of Endocrinology, Narodni 8, Prague 1, 116 94, Czech Republic, E-mail: MH: mhill@endo.cz

Prof. Vojtech Hainer, M.D., Ph.D.

Institute of Endocrinology, Narodni 8, Prague 1, 116 94, Czech Republic, E-mail: VH: vhainer@endo.cz
